# Experimental Study on the Microstructure and Tribological Properties of Laser-Clad Ni60-WC Composite Coatings

**DOI:** 10.3390/ma17184638

**Published:** 2024-09-21

**Authors:** Yupeng Cao, Kai Yan, Weidong Shi, Rui Zhou, Bin Li, Jiaxin Qin

**Affiliations:** 1 School of Mechanical Engineering, Nantong University, Nantong 226019, China; cyp19812004@ntu.edu.cn (Y.C.); 2210310032@stmail.ntu.edu.cn (K.Y.); qin2109310035@163.com (J.Q.); 2 School of Information Science and Technology, Nantong University, Nantong 226019, China; 3 School of Mechanical Engineering, Nantong Institute of Technology, Nantong 226001, China; libin19@ntit.edu.cn

**Keywords:** laser cladding, composite coatings, microstructure, tribological performance

## Abstract

To address the wear issues faced by the leg components of offshore platforms in harsh marine conditions, a Ni60-WC composite coating was fabricated on the surface of E690 high-strength steel using laser cladding. The microstructure, elemental distribution, microhardness, and tribological properties of the composite coating were characterized and tested using XRD (X-ray diffraction), SEM (scanning electron microscopy), EDS (energy-dispersive spectrometry), a microhardness tester, and a multifunctional tribometer. The study focused on the microstructure and tribological properties of the Ni60-WC composite coating. The results show that the composite coating primarily consists of γ-(Fe, Ni), WC, W2C, M23C6, and M6C phases, with cellular and dendritic structures at the top. WC and W2C, along with M23C6 and M6C, are precipitated from the W and C elements. The average hardness of the composite coating reached 569.5 HV, representing a 103% increase over the substrate hardness. The prepared composite coating exhibited a 32.6% increase in corrosion potential compared to the substrate. Additionally, the corrosion current density was reduced by 62.0%, indicating a significant enhancement in the corrosion resistance of the composite coating. The friction coefficient of the composite coating was reduced by 17.4% compared to the substrate, and wear volume was reduced by 79%, significantly enhancing the tribological performance of the coating due to reduced abrasive wear and fatigue wear.

## 1. Introduction

The leg components of offshore platforms, essential structural elements in marine engineering, face severe wear and corrosion due to long-term exposure to harsh marine environments. Laser cladding technology, which directly deposits material powder onto the surface of a workpiece to form a metallurgically bonded coating, has been a focus of recent research [1,2]. This technology significantly improves the substrate’s properties and has been widely applied in the rapid prototyping and remanufacturing of parts [3,4]. 

Adding high-hardness compounds to the cladding layer effectively enhances its wear resistance [5,6]. Xu [7] observed a transition from coarse dendritic grains to denser equiaxed grains in the coatings they prepared, significantly improving wear resistance; Zhu [8] and Li [9] successfully fabricated composite coatings with good hardness on TC4 alloy and 35CrMo steel, demonstrating their potential in engineering applications. Ni-WC composite coatings are favored for their excellent hardness, wear resistance, and corrosion resistance, particularly in conditions requiring high wear and corrosion resistance [10,11,12,13]. Tan [14] prepared Ni-WC composite coatings with good hardness as well as wear resistance on the surface of 304 steel, and Li [15] applied WC composite coatings to agricultural machinery, which effectively improved the quality of metal-based coatings. However, research on the application of Ni-WC composite coatings prepared by laser cladding in marine engineering is still relatively scarce. Therefore, using laser cladding to fabricate and study the microstructure and tribological properties of Ni-WC composite coatings on offshore platform legs is of great industrial value for the marine engineering field.

In this study, Ni-WC composite coatings were prepared on the surface of an E690 high-strength steel substrate using optimized laser cladding parameters. The microstructure, morphology, elemental distribution, and hardness of the composite coatings were characterized and tested using X-ray diffraction (XRD), scanning electron microscopy (SEM), energy-dispersive X-ray spectroscopy (EDS), and microhardness testing. Electrochemical corrosion tests were conducted using an electrochemical workstation to simulate marine corrosion environments and assess the corrosion resistance of the coatings. Friction and wear experiments were performed under oil-deprived conditions to evaluate the tribological performance of the composite coatings. High-resolution confocal microscopy and SEM were used to observe the failure characteristics on the sample surfaces and explore the effect of different normal loads on the wear behavior of the repair layers. This study reveals the wear resistance mechanisms of the composite coatings, expanding the application of green remanufacturing technologies in marine engineering, with significant theoretical and engineering implications. 

## 2. Experimental Materials and Methods

### 2.1. Experimental Materials

**Figure 1:**
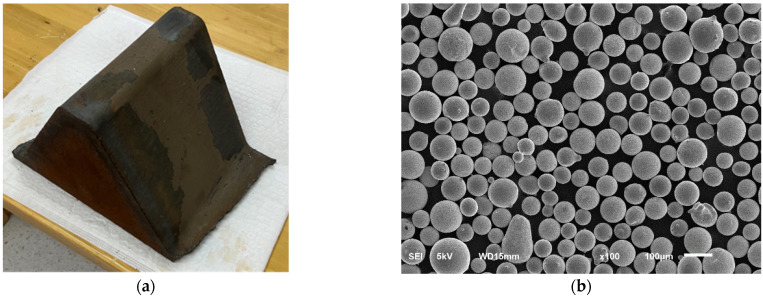
Experimental materials: (**a**) E690 steel single tooth; (**b**) SEM image of the powder.

In the laser cladding experiment, the substrate material used was American-imported E690 high-strength steel, sourced from the rack and pinion lifting system of Zhenhua Heavy Industries’ offshore platform legs. The image below shows a single tooth cut from the rack. Samples were machined from this material using wire cutting into dimensions of 50 mm × 50 mm × 10 mm. To ensure surface cleanliness, the sample surfaces were polished with sandpaper and then cleaned in an ultrasonic cleaner using 95% ethanol for 15 min. The cladding material selected was Ni60-WC spherical powder, custom-prepared by Guangdong Chengfeng (Zhaoqing, China). The morphology of the powder is shown in Figure 1, with a WC content of 65%. The Ni-based alloy powder had a particle size range of 50–150 μm, while the WC particles ranged from 30 to 105 μm. The chemical composition of the E690 high-strength steel substrate and the Ni-based powder is detailed in Table 1.

### 2.2. Experimental Methods

The equipment used for the laser cladding experiments was provided by Nanjing Zhongke Yuchen Laser Technology Co., Ltd., Beijing, China, with a laser wavelength of 1100 nm. The equipment can precisely set and control parameters such as scanning speed, powder feed rate, and laser power. Based on previous research by the research group, optimized laser cladding process parameters were used to prepare the Ni60-WC composite coating on the surface of the sample. The optimized laser cladding process parameters are shown in Table 2. The laser focuses on the surface of the substrate. Argon gas was used as both the shielding and powder carrier gas, with a shielding gas flow rate of 7.5 L/min and a powder carrier gas pressure flow rate of 10 L/min.

X-ray diffraction (XRD) was performed using an Ultima IV diffractometer (Rigaku, Tokyo, Japan) in continuous scanning mode, with a 2θ range of 5° to 120° and a scanning speed of 4°/min. The microstructure and elemental distribution of the laser-clad Ni60-WC composite coatings were observed using a field-emission scanning electron microscope (JSM-7000F, JEOL, Tokyo, Japan). The cross-sectional SEM images were captured at a magnification of 2500×, while the wear morphology SEM images were taken at a magnification of 500×.

The microhardness of the surface and cross-section of the composite coatings were measured using a digital Vickers microhardness tester (TMVS-1, Beijing Time High Technology Ltd., Beijing, China).

Microhardness measurements were taken every 150 μm along the depth of the sample section, with five points measured at each depth to obtain an average value. To avoid conflict in the results, the distance between two consecutive indentations was kept greater than three times the diagonal length of the indentation.

Electrochemical corrosion experiments were conducted using an electrochemical workstation (CHI660C, Chenhua, Shanghai, China) and a conventional three-electrode system to investigate the corrosion resistance of the samples. A 3.5% NaCl solution was used as the electrolyte. During the electrochemical corrosion tests, the potential polarization curve was measured with a test duration of 300 s. The initial potential was set at −1.5 V and the final potential at 0 V, with a scan rate of 0.001 V/s and a sampling interval of 0.01 s. Finally, impedance spectra were obtained with the initial potential set at −1.5 V, a high frequency of 100 kHz, a low frequency of 0.1 Hz, and an amplitude of 0.005 V.

A friction test machine (MFT-5000, Rtec-Instruments, San Jose, CA, USA) was used for the friction and wear experiments; the reciprocating module was chosen to be silicon nitride balls with a diameter of 6 mm and a hardness of 1500–1900 HV, and the friction test parameters are shown in Table 3. Subsequently, high-resolution confocal microscopy (NanoFocus usurf, Oberhausen, Germany), SEM, and EDS were used to observe the failure structural features and elemental changes on the worn surface.

## 3. Results and Discussion 

### 3.1. Microstructural Analysis of Laser-Clad Composite Coating

#### 3.1.1. SEM Analysis

**Figure 2:**
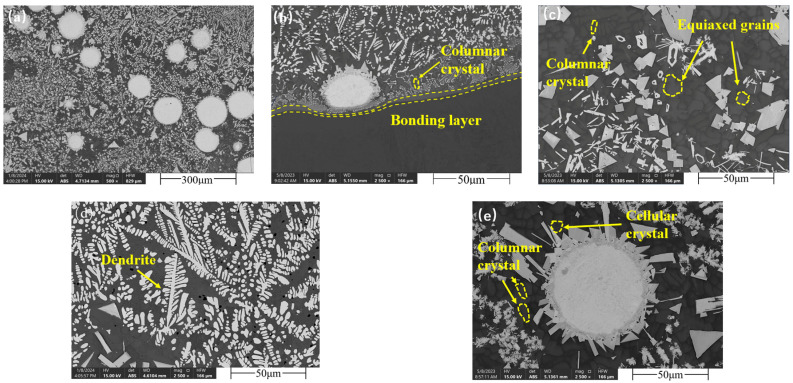
SEM images of the composite coating: (**a**) cross-sectional morphology of the composite coating; (**b**) bottom of the composite coating; (**c**) middle of the composite coating; (**d**) top of the composite coating; (**e**) WC particles in the composite coating.

The SEM morphology of the composite coating is depicted in Figure 2. Observations of the coating cross-section in Figure 2a reveal the absence of apparent pores or cracks. The microstructure during the laser cladding solidification process is primarily influenced by the temperature gradient (G), solidification rate (R), and cooling rate (dT/dt). The G/R ratio determines the characteristics of the microstructure, while dT/dt affects the scale of the microstructure [16]. At the onset of pool solidification, rapid cooling at the base of the substrate results in a high G/R, producing a strong cooling effect. As shown in Figure 2b, this cooling effect leads to the formation of a thin planar crystal layer at the interface of the clad layer, known as the solid-solution bonding layer. Additionally, the columnar crystals near the interface enhance the composite coating’s plasticity and creep resistance. The absence of large-angle grain boundaries indicates that the metallurgical bond between the coating and the substrate is more uniform and robust, thereby improving the coating’s adhesion and bonding quality [17]. Figure 2c illustrates that a large number of columnar and equiaxed crystals form in the middle part of the composite coating due to the formation of the solid-solution bonding area. The thermal diffusion in the melt pool slows down, reducing the temperature gradient G at the solid–liquid interface and the solidification rate R, thereby slowing the advance of the solid–liquid interface. The increased undercooling allows nuclei to grow in multiple directions, overcoming the directional growth advantage of columnar crystals and promoting the formation of equiaxed crystals. Equiaxed crystals have a higher grain boundary density and a more uniform grain structure. A low G/R ratio also results in grain refinement with more small grains being formed, which enhances the material’s strength and toughness [18].

As shown in Figure 2d, a large number of dendrites form at the top of the composite coating. This occurs because the direct heating by the laser and cooling by the protective gas create a significant temperature gradient at the top of the coating, causing nuclei to grow rapidly along the direction of heat flow and form dendritic grains [19,20]. In the WC-containing composite coating, these dendrites effectively disperse and stabilize hard-phase particles, forming a network structure of the reinforcing phase, which further enhances the coating’s hardness and wear resistance. The microstructure around WC particles grows perpendicularly to their boundaries, forming columnar and cellular structures. As shown in Figure 2e, the grains around the WC particle agglomeration area are primarily cellular with some columnar crystals, and the formation of numerous cellular crystals contributes to the stability of the composite coating [21].

#### 3.1.2. EDS Elemental Analysis

**Figure 3:**
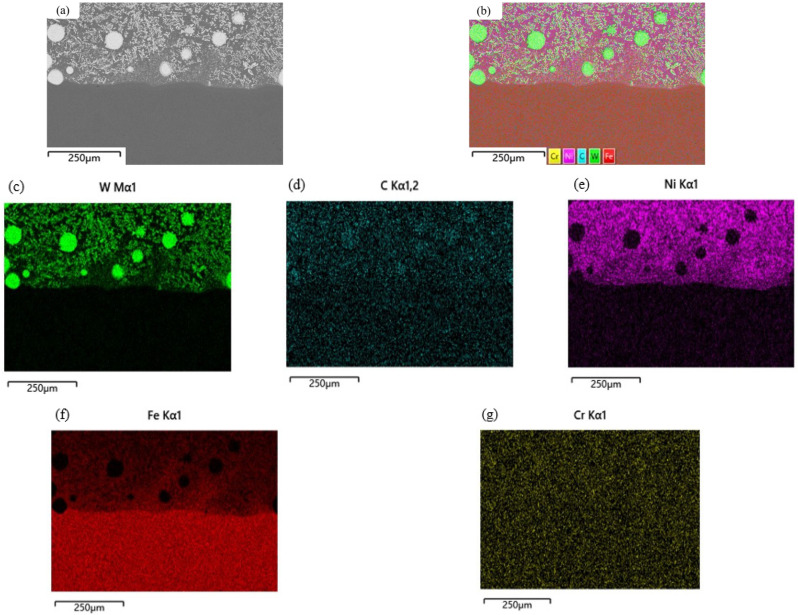
EDS surface scan results of the composite coating cross-section: (**a**) area of the composite coating scanned; (**b**) total elemental distribution scan results; (**c**) W element distribution; (**d**) C element distribution; (**e**) Ni element distribution; (**f**) Fe element distribution; (**g**) Cr element distribution.

To further investigate the elemental distribution of the composite coating, EDS area and line scans were performed on the composite coating region, focusing on the Ni-WC composite coating interface. The results are shown in Figure 3, with Figure 3a representing the selected area for elemental analysis. Figure 3b reveals that the composite coating is primarily composed of Cr, Ni, Fe, W, and C elements. Figure 3c,d demonstrate that a significant amount of W is closely associated with WC particles, while the rest of the W is evenly distributed within the composite coating around the WC particles, showing no apparent diffusion trend towards the substrate. In areas without WC, the W content is relatively low. C elements, on the other hand, are evenly distributed throughout the entire composite coating without significant aggregation, differing from the distribution characteristics of the W elements. This difference arises because W elements originate solely from partially decomposed WC particles, while C elements come from both the Ni alloy powder and WC particles [22].

Combined with the cross-sectional SEM image in Figure 2, it is evident that the content of W and C elements in the dendrites is significantly higher than in the composite coating matrix, indicating that W and C elements participate in the precipitation process of hard carbides. Notably, C elements are involved in the precipitation of carbides such as M23C6 and M6C. The uniform distribution of C elements and the formation of carbide phases contribute to the improved hardness and wear resistance of the composite coating. 

**Figure 4:**
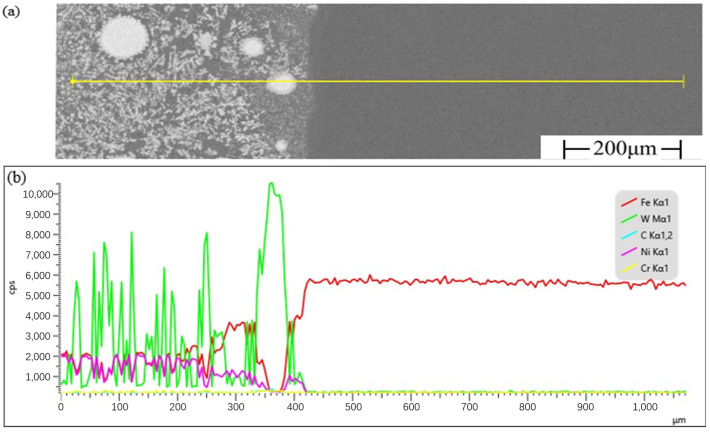
Line scan area and results of the composite coating cross-section: (**a**) line scan area of the composite coating; (**b**) line scan results of the composite coating.

The line scan region and results of the composite coating cross-section are shown in Figure 4, the yellow line in Figure 4a represents the line scan area of the composite coating. Observations from Figure 4b reveal the presence of a transition area between the composite coating and the substrate, where the Ni element content decreases while the Fe element content increases. Combined with the results from Figure 3e–g, it is evident that Ni and W elements are detected at the top of the substrate, indicating the diffusion of Fe, Ni, and W elements between the composite coating and the substrate. The Fe element diffuses from the substrate to the composite coating, while Ni and W elements diffuse from the composite coating to the substrate, although the extent and range of diffusion are relatively small. Moderate diffusion in the transition area can improve the interface’s bonding strength. Additionally, the transition area can effectively reduce interface defects, enhancing the bonding performance of the composite coating with the substrate [23]. In the vicinity of WC phases, the content of Ni and Fe elements drops sharply, while the W element content rises sharply, and the changes in C and Cr elements from the Ni-base binder phase to the WC reinforcing phase are not very pronounced, indicating a metallurgical bond between the WC phase and the Ni-base binder phase [24].

#### 3.1.3. XRD Spectrum Analysis

**Figure 5:**
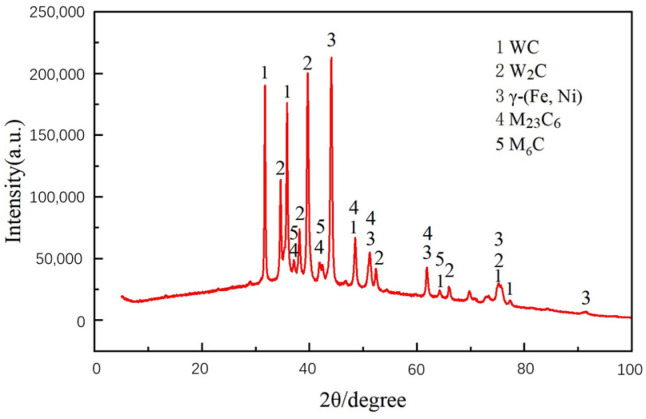
XRD spectrum of the composite coating.

The XRD spectrum of the Ni-WC-clad layer prepared in the experiment is shown in Figure 5. Combined with the EDS elemental distribution from Figure 3 and Figure 4, it is evident that the composite coating is primarily composed of γ-(Fe, Ni), WC, W2C, M23C6, and M6C phases. The base phase is γ-(Fe, Ni), with the reinforcing phases mainly consisting of WC, W2C, and various carbides. Additionally, the (Fe, Ni) solid solution supports these hard phases.

In summary, WC particles melt and decompose under laser action to form W2C. As 2.time progresses, the precipitation of W and C elements increases, and these elements dissolve in the Ni alloy to form a solid solution, thereby providing solid-solution strengthening [8]. Simultaneously, carbides such as M23C6 and M6C form in the melt pool, acting as nucleation sites during crystallization to promote the refinement of the composite coating structure. This results in both solid-solution strengthening and fine-grain strengthening, thereby enhancing the overall hardness of the composite coating.

Through the use of SEM, XRD, and EDS area and line scans, it is evident that the prepared Ni-WC composite coating exhibits high quality. The WC particles form a strong bond with the substrate and alloy under laser treatment, significantly enhancing the coating’s hardness. The diffusion of elements in the transition area between the coating and the substrate further strengthens the bond, resulting in a more stable and reliable coating.

### 3.2. Microhardness Distribution across the Composite Coating Cross-Section

**Figure 6:**
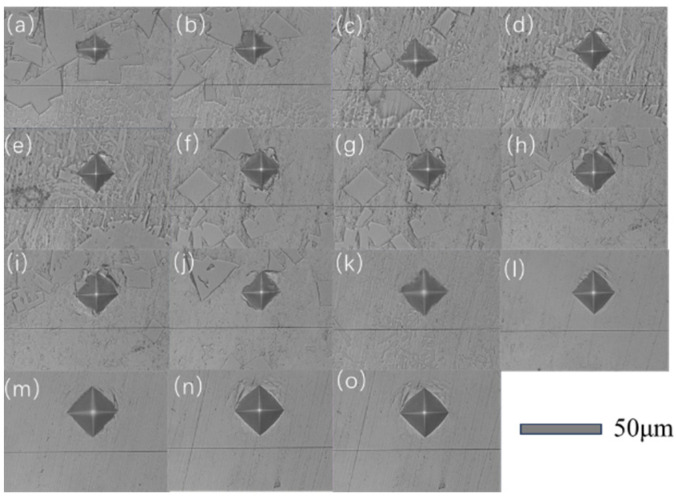
Microhardness indentations at different depths of the composite coating cross-section. (**a**–**o**) is a measurement image taken every 150 μm from the top to the bottom of the coating section.

**Figure 7:**
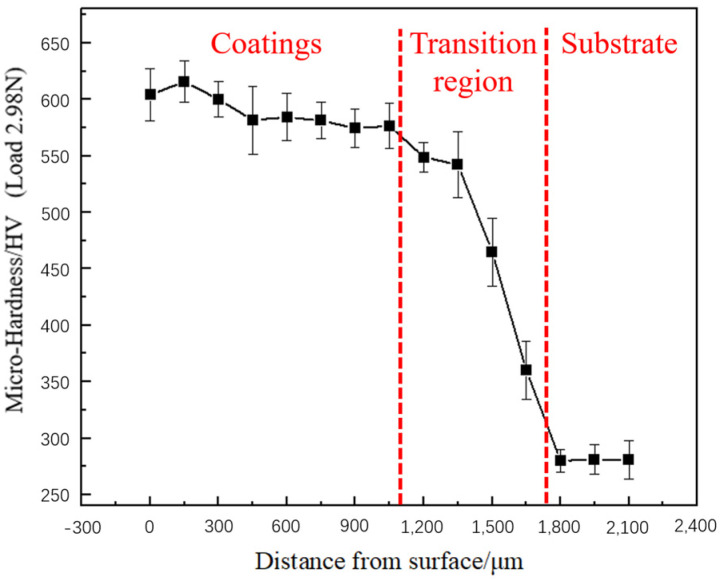
Microhardness distribution across the composite coating cross-section.

The microhardness indentations at different depths of the composite coating cross-section are shown in Figure 6, with the test results displayed in Figure 7. The microhardness of the composite coating cross-section is mainly divided into three areas: the composite coating area, the transition area, and the substrate area. The average hardness of these areas is 569.5 HV, 498.52 HV, and 280.5 HV, respectively, with the composite coating hardness showing a 103% increase compared to the substrate. In the composite coating area, as the depth increases, the bottom hardness decreases by approximately 4.6% compared to the top. Observations from Figure 2d (the cross-sectional SEM image of the top of the coating), indicate that during processing, the top of the coating, directly affected by the laser, exhibits a large temperature gradient, leading to the formation of numerous dendrites containing many grain boundaries. This generally hinders the movement of dislocations, thereby enhancing the material’s hardness to some extent [19]. Observations of the hardness changes in the transition area reveal that the hardness decreases from 569.5 HV to 498.52 HV, but the average hardness still shows a 77.7% increase compared to the substrate. This increase is due to the coarsening of grains in the transition area under laser action, reducing the material’s hardness [25]. Overall, this leads to a characteristic where the composite coating area exhibits higher hardness at the top and lower hardness at the bottom.

### 3.3. Study on the Electrochemical Corrosion Performance of the Composite Coating

**Figure 8:**
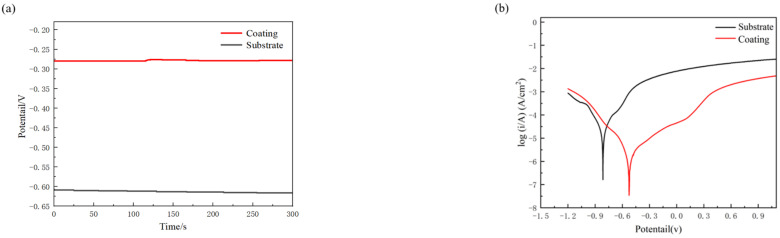
Open circuit potential and polarization curves of the samples: (**a**) open circuit potential; (**b**) polarization curve.

To enhance the study of composite coatings’ performance in marine environments, experiments were conducted to analyze the corrosion resistance of the coatings. The high concentration of chloride ions and complex electrochemical conditions in marine environments accelerate the corrosion process. Therefore, electrochemical corrosion tests were used to simulate these corrosive conditions, providing a rapid assessment of the coatings’ long-term corrosion resistance in actual marine environments [26]. The relationship between open circuit potential and time for samples in a 3.5% NaCl solution is shown in Figure 8a. The open circuit potential indicates the tendency for electrochemical corrosion, with lower potentials suggesting poorer corrosion resistance. The open circuit potential of the E690 high-strength steel substrate was −0.64 V vs. SCE, while the composite coating shifted positively to −0.28 V vs. SCE, an improvement of 57.6% over the substrate. This indicates a significant reduction in the corrosion tendency of the composite coating, demonstrating a marked improvement in corrosion resistance compared to the substrate.

The potentiodynamic polarization curves of the samples in the corrosive solution are shown in Figure 8b. The corrosion potential and corrosion current density represent the corrosion rate of the samples; a lower corrosion potential and a higher corrosion current density indicate a faster corrosion rate. As observed in Figure 8b, the corrosion potential of the substrate is −0.814 V, with a corrosion current density of 0.129 μA/cm^2^. In contrast, the prepared composite coating exhibits a corrosion potential of −0.548 V, which is an increase of 32.6%. The corrosion current density of the composite coating is 0.049 μA/cm^2^, representing a 62.0% reduction. These results demonstrate that the prepared composite coating has a lower corrosion rate and significantly enhanced corrosion resistance.

**Figure 9:**
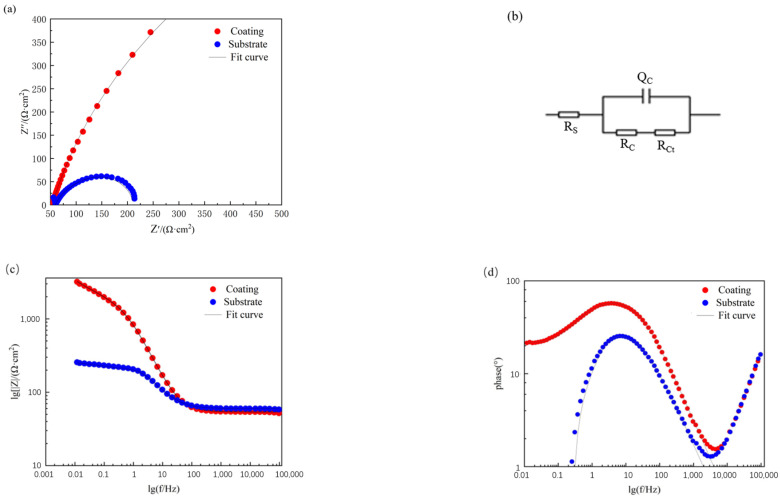
Impedance spectra of the samples: (**a**) Nyquist plot of the samples; (**b**) fitting circuit; (**c**) Bode magnitude plot of the samples; (**d**) Bode phase angle plot of the samples.

Figure 9 shows the impedance spectra of laser cladding in a 3.5 wt.% NaCl solution. As observed in Figure 9a, both the E690 high-strength steel substrate and the composite coating exhibit Nyquist plots characterized by a single capacitive semicircle in the high-frequency region. The corrosion rate is closely related to the radius of the capacitive arc; a larger radius indicates greater corrosion resistance. The figure clearly demonstrates that the radius of the capacitive arc for the composite coating is significantly larger than that of the substrate, confirming the enhanced corrosion resistance of the composite coating.

By fitting the electrochemical impedance spectra with an equivalent circuit, the corrosion resistance of the coating can be more accurately assessed. The fitting circuit used is shown in Figure 9b, where Rs represents the solution resistance, R_c_ denotes the resistance of the composite coating, and R_ct_ stands for the charge transfer resistance at the interface. Figure 9c,d display the electrochemical corrosion Bode plots, which include two types of curves: the |Z|–frequency curve, where low-frequency Log|Z| corresponds to Rct (charge transfer resistance) and high-frequency Log|Z| corresponds to R_c_ (solution resistance); and the phase angle–frequency curve. Both impedance values for the composite coating increase, and the phase angle plateau in the mid-frequency range is broadened, indicating a continuous improvement in the corrosion resistance of the composite coating.

### 3.4. Tribological Performance Study of the Laser-Clad Composite Coating

#### 3.4.1. Effect of Normal Load on Friction Performance

**Figure 10:**
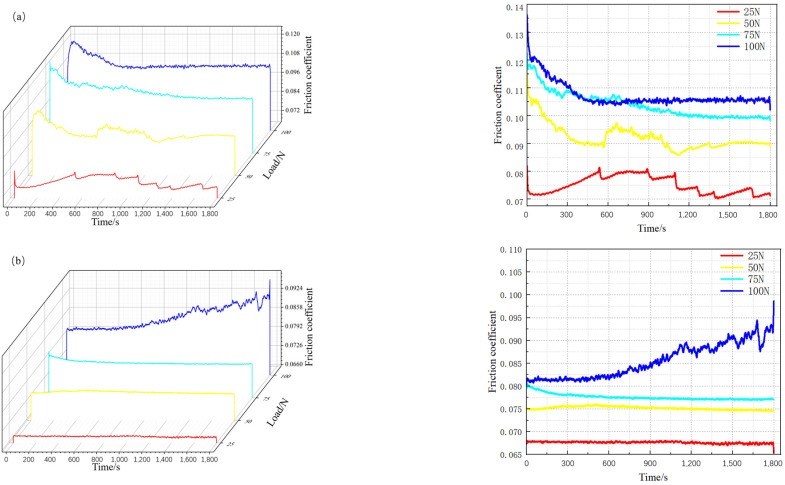
Three-dimensional and two-dimensional friction coefficient curves of the substrate and composite coating under different normal loads: (**a**) substrate; (**b**) composite coating.

Figure 10 shows the friction coefficient curves of the E690 high-strength steel substrate and the Ni60-WC composite coating under different loads. Observing Figure 10a, which depicts the friction coefficient curves of the E690 high-strength steel substrate under varying loads, it is evident that the friction coefficient increases with the increase in load and exhibits less fluctuation. This behavior is attributed to the fact that, at lower loads, the actual contact area between the surfaces is smaller, leading to intermittent stick–slip phenomena during friction, which causes greater fluctuations in the friction coefficient. Conversely, at higher loads, the actual contact area increases, allowing the larger contact area to distribute the load more evenly, resulting in a more stable friction process and a more consistent friction coefficient curve.

Figure 10b shows that the overall friction coefficient curve of the Ni-WC composite coating follows a similar trend to that of the substrate under varying loads, with the friction coefficient increasing as the load increases. However, compared to the E690 substrate, the composite coating exhibits smaller fluctuations in the friction coefficient curve under the same load. This stability is due to the high hardness of the WC composite coating, which maintains a stable surface morphology under load, stabilizing the friction coefficient curve.

Observing Figure 10b and comparing the 100 N load with lower loads, it can be seen that at a load of 100 N, the friction coefficient curve remains relatively stable in the initial phase. However, as time progresses, the friction coefficient increases sharply. This sudden increase can be correlated with the indentations and wear volume. The sharp fluctuations in the friction coefficient are likely due to the formation of wear particles between the coating and the friction ball during the friction process. These wear particles may roll or slide between the contact surfaces, causing a transition from two-body wear to three-body abrasive wear, which in turn leads to an abrupt rise in the friction coefficient of the composite coating [27,28]. 

**Figure 11:**
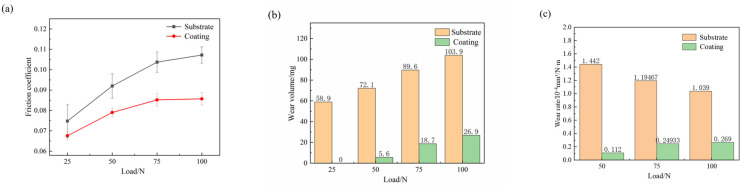
Friction coefficients and wear volumes of the substrate and composite coating under different normal loads: (**a**) friction coefficients; (**b**) wear amounts; (**c**) wear rates.

Observations from Figure 11 of the E690 high-strength steel substrate and the Ni60-WC composite coating under different loads show that, as the normal load increases, both the friction coefficient and wear volume of the substrate and composite coating increase. However, Figure 11a clearly demonstrates that the friction coefficient of the composite coating is significantly lower than the E690 substrate. Under a 50 N load, the substrate’s friction coefficient is approximately 0.0920, while the composite coating’s friction coefficient is lower at 0.0790, representing a 14.3% reduction. This highlights the composite coating’s significant advantage under low loads.

As the load increases to 100 N, the substrate’s wear volume increases to 103.9 mg, while the composite coating’s wear volume is only 26.9 mg, a 74.1% reduction compared to the substrate, further demonstrating the excellent wear resistance of the Ni60-WC composite coating.

Based on the observation of Figure 11c, it is evident that under a load of 25 N, the wear rate of the substrate is 1.442 × 10^−6^ mm^3^/(N·m), while the wear rate of the composite coating at the same load is only 0.112 × 10^−6^ mm^3^/(N·m). Even under loads of 75 N and 100 N, the wear rate of the composite coating remains significantly lower than that of the substrate material. This improvement in wear resistance is attributed to the decomposition of WC particles during the laser cladding process, leading to the formation of carbides that enhance the coating’s wear resistance. Consequently, the prepared composite coating effectively improves the material’s wear resistance.

These data indicate that although the friction coefficients and wear volumes of both the E690 high-strength steel substrate and the composite coating increase with load, the composite coating consistently exhibits lower friction coefficients and wear volumes under the same load conditions. This underscores the composite coating’s excellent wear resistance and improved surface properties under high-load conditions. This phenomenon can be attributed to the high hardness and superior wear resistance of WC particles, as well as the laser cladding process, which forms a strong bond between the coating and the substrate, further enhancing the wear resistance of the composite coating.

#### 3.4.2. Wear Morphology Analysis

**Figure 12:**
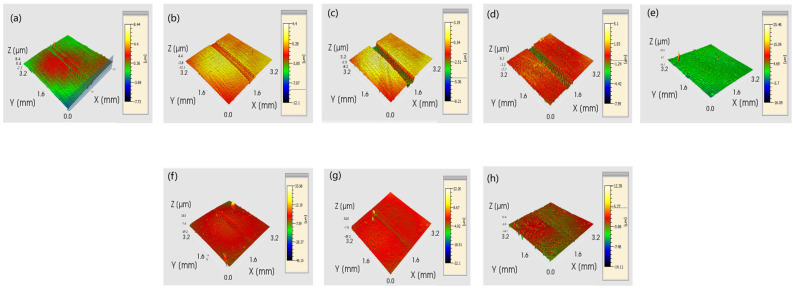
Surface three-dimensional morphology of the substrate and composite coating after friction and wear under different normal loads: (**a**) 25 N substrate; (**b**) 50 N substrate; (**c**) 75 N substrate; (**d**) 100 N substrate; (**e**) 25 N composite coating; (**f**) 50 N composite coating; (**g**) 75 N composite coating; (**h**) 100 N composite coating.

**Figure 13:**
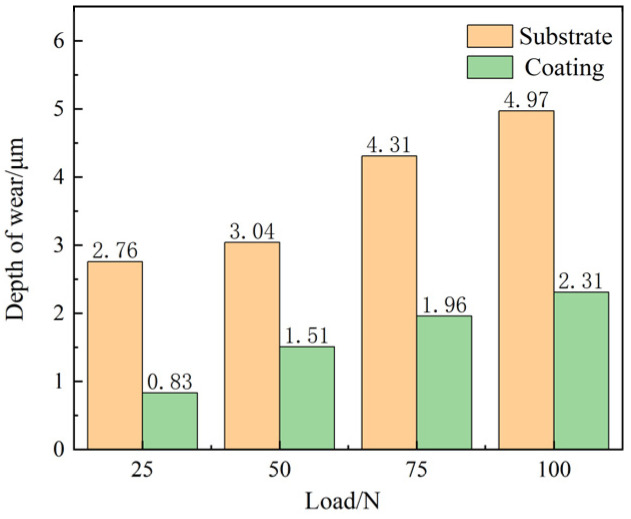
Wear depths of the substrate and composite coating under different normal loads.

Figure 12 shows the surface three-dimensional morphology of the E690 high-strength steel substrate and the Ni60-WC composite coating after friction and wear under different normal loads. When the normal load is 25 N, noticeable wear marks appear on the substrate surface, with a wear depth of 2.76 μm, while no wear marks are observed on the composite coating surface under the same pressure. The wear depths under 50 N, 75 N, and 100 N load conditions are shown in Figure 13. The substrate’s wear depths are 3.04 μm, 4.31 μm, and 4.97 μm, respectively, while the composite coating’s wear depths are 1.51 μm, 1.96 μm, and 2.31 μm, respectively, showing reductions of 50.3%, 54.5%, and 53.5% compared to the substrate.

As the load increases, the wear depths of both the substrate and composite coating show an increasing trend, but under the same load conditions, the wear depth of the composite coating is significantly less than that of the substrate. This demonstrates that the prepared composite coating has higher hardness and wear resistance.

**Figure 14:**
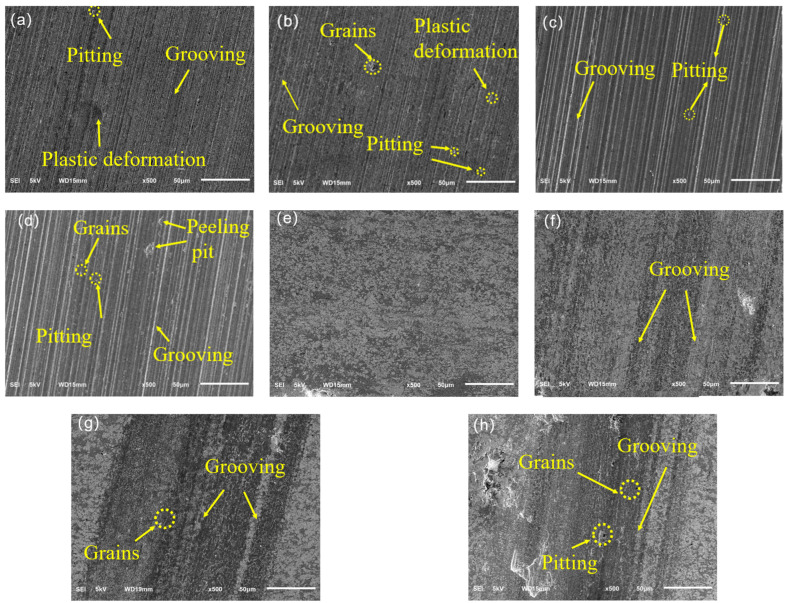
Surface SEM morphology of the E690 high-strength steel and composite coating after wear under different normal loads: (**a**) 25 N substrate; (**b**) 50 N substrate; (**c**) 75 N substrate; (**d**) 100 N substrate; (**e**) 25 N composite coating; (**f**) 50 N composite coating; (**g**) 75 N composite coaching; (**h**) 100 N composite coating.

The surface SEM morphology of the E690 high-strength steel and composite coating after wear under different normal loads is shown in Figure 14. When the normal load is 25 N, the substrate exhibits clear signs of plastic deformation, with numerous plowing grooves and a few pitting holes appearing on the surface, indicating significant abrasive and fatigue wear. In contrast, no wear marks are found on the composite coating surface. As the normal load increases to 50 N, the number and width of plowing grooves on the substrate surface increase, intensifying abrasive wear, while the composite coating surface only shows a few narrower plowing grooves, indicating slight abrasive wear. 

At a normal load of 75 N, the depth and width of the plowing grooves on the substrate surface further increase, accompanied by an increase in the number of pitting holes, indicating intensified abrasive and fatigue wear. The composite coating surface also shows increased an width and depth of the plowing grooves, with some fine abrasive particles observed within the grooves, indicating intensified abrasive wear.

When the normal load reaches 100 N, the number of pitting holes on the substrate further increases, with some areas showing adhesive peeling pits, indicating intensified fatigue and adhesive wear. The composite coating also shows a few pitting holes, indicating fatigue wear. 

As the load increases, both the abrasive and fatigue wear of the E690 high-strength steel substrate and the composite coating increase. This is mainly due to the increased normal load, which causes higher contact stress on the surface, leading to more severe plastic deformation and stress concentration. This results in more abrasive particles sliding and cutting on the material surface, intensifying the formation and propagation of fatigue cracks and the wear process.

Compared to the substrate, the composite coating experiences significantly reduced abrasive and fatigue wear under the same load, indicating a marked improvement in the wear resistance of the composite coating.

#### 3.4.3. The Wear Mechanism of the Composite Coating

**Figure 15:**
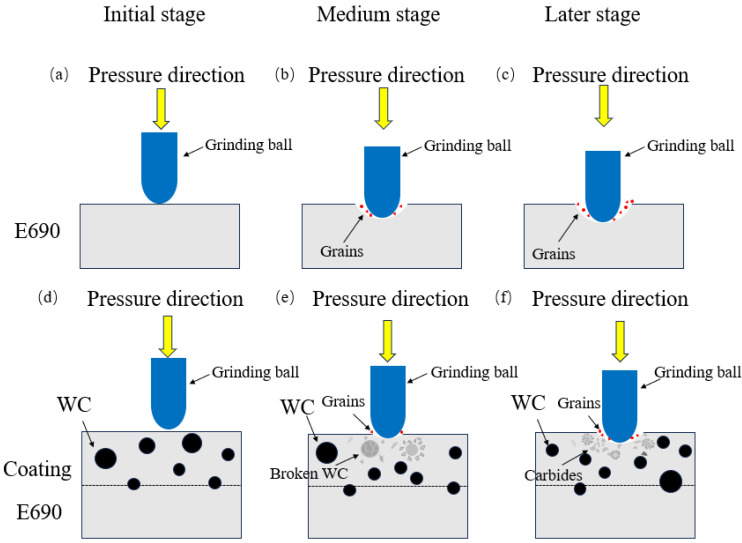
Wear mechanism of substrate and Ni60 WC composite coating.

The wear mechanisms of the E690 high-strength steel and Ni60-WC composite coatings during surface manufacturing are illustrated in Figure 15. In the early stages of wear, the substrate undergoes plastic deformation under the applied load. As friction continues into the mid-stage, significant abrasive wear occurs on the substrate, resulting in the formation of grooves and the detachment of some material, which enters the frictional process. In the later stages of wear, the detached material further enters the contact interfaces, transitioning from two-body abrasive wear to three-body abrasive wear, which significantly intensifies wear. This process leads to the formation of numerous wear particles and spalling pits in some regions.

For the composite coating, the surface hardness of the Ni-based matrix is higher than that of the substrate, making plastic deformation under load difficult during the initial stages of friction. In the mid-stage of wear, only a small amount of material detaches to form wear particles. In the later stages, as the WC particles possess significantly higher hardness compared to the Ni-based alloy, the Ni matrix undergoes wear, exposing the WC particles. This exposure helps to inhibit further wear progression. Over time, some WC particles develop cracks and eventually fracture. These fractured WC particles interact with the Ni-based alloy, forming a blocky structure through alloying, which further suppresses wear. As a result, the composite coating demonstrates significantly improved hardness and wear resistance compared to the substrate.

## 4. Conclusions

This paper presents the preparation of Ni60-WC composite coatings on the surface of E690 high-strength steel using laser cladding. The structural properties of the composite coatings were studied using XRD, SEM, EDS, and a microhardness tester. The wear mechanisms and tribological performance of the composite coatings were also investigated through wear experiments, leading to the following conclusions:

1. The composite coating primarily consists of γ-(Fe, Ni), WC, W2C, M23C6, and M6C phases, with the (Fe, Ni) solid solution supporting these hard phases. A transition area forms in the bonding region between the composite coating and the substrate, where cellular and equiaxed crystals at the top of the composite coating contribute to fine-grain strengthening.
2. In the transition area at the interface between the composite coating and the substrate, diffusion behaviors of Fe, Cr, and Ni elements occur, effectively improving the bonding performance of the coating. W and C elements from the decomposition of WC actively participate in the precipitation process of hard carbides, enhancing the hardness of the composite coating. The average hardness of the composite coating reaches 569.5 HV, representing a 103% increase compared to the substrate. The corrosion potential of the composite coating increased by 32.6% compared to the substrate. The corrosion current density decreased by 62.0%. These results indicate that the fabricated composite coating exhibited a reduced corrosion rate and a significant improvement in corrosion resistance.
3. As the normal load increases, both the friction coefficient and wear volume of the substrate and composite coating increase. However, under the same normal load, the friction coefficient of the composite coating is reduced by 17.4%, the wear volume is reduced by 79%, and the wear depth is reduced by 54.4%, significantly reducing abrasive wear and fatigue wear. This improvement greatly enhances the tribological performance of the composite coating.
4. Research on the Ni60-WC composite coating still lacks sufficient investigation into whether new phases are generated after wear, whether stress distribution changes, and whether there is element migration or consumption. Future studies could focus on the relationship between the microstructure and performance of the coating, as well as further enhancing the understanding of its corrosion resistance. This would contribute to improving the overall performance of the composite coating, expanding its range of applications, and providing more reliable and durable material solutions for marine engineering.

## Figures and Tables

**Table 1:** Chemical composition of cladding substrate and powder (mass fraction, %).

Element	C	Si	Mn	P	S	Cr	Ni	Mo	V	Cu	Fe
Substrate	0.15	0.50	1.52	0.03	0.01	1.50	3.60	0.70	0.06		Bal.
Powder	8	4				1	60			17.5	9.5

**Table 2:** Laser cladding parameters.

Laser Power (W)	Laser Pulse Width (ns)	Spot Diameter (mm)	Powder Feed Rate (r/min)	Laser Scanning Speed (mm/s)
2200	20	3	0.7	7

**Table 3:** Friction and wear test parameters.

Frequency (Hz)	Normal Load (N)	Time (S)	Temperature (°C)
4	25, 50, 75, 100	1800	25

## Data Availability

The original contributions presented in the study are included in the article, further inquiries can be directed to the corresponding author.
